# Supplementary material for: Chromothripsis during telomere crisis is independent of NHEJ, and consistent with a replicative origin
Source: Genome Res. 2019 May;29(5):737–49. doi: 10.1101/gr.240705.118 (PMC6499312; doi:10.1101/gr.240705.118)
Supplement: Supplemental Material [file supp_gr.240705.118_Supplemental_file_1.zip › contigs/annotated_contigs/DB82/contig.2.DB82_length_650_mean_cov_11.7723076923.docx]

**DB82_length_650_mean_cov_11.7723076923**

```
TCTGAAACTTACTCTCTCTTTCCTGATCAATATATGCCATCATTTGGTGAACATACAGAAATTTATTTACCATAGACAACATAATATAC
 >chr4:19976062-19976432 - E=6e-211
TTTAAAAGTATATTAGTTAGAGATAGCCAAAATATTCAGGAAGTTATTACTCATTTATTTTTAATTTTTCCTAGTATTAAGTTAGGCCA

TGTGAAATCGCAATTTTTGTAGATACAAAAAAAGATGAATAAAGGCAATTTCATATTGCCCAGCTATTTAAATGAGCCTCTTTCTCTCA

AGTTTTCTGTCATTTTGAAAAAAAATTGTTTGAGACAAAAAATGTTTTGTTCAATTTTCAGCCATAACAACCTTGCCTAGGTTACTGTT

TTGTACTCTCCCTA|GAGAAAGTCC|TAGAGAGGACTTTCATGTCTAGAAAAAAATCTGTATAGTAAAGGAATATGTAGCTCTGTGTAT
                         >chr4:19974640-19974910 - E=1e-144
ATCTCCTTAGTAGTTCTTAGCACAGTTGCAGTTTTACACATTTGTGTAGTTAATTGATTGATATCTGCCTTTCCAATCAAGTATTGAAA

TATTATGAATTTAGTGGTCTAGCTACTTCACTACCTGGAATAGTGCGTGGAACATCATACAGATTCAATGACATTTGTTGACTGAGTAA

ATAAATATAAAGATACTAGAAGGAGGTGT
```
